# Translation, cultural adaptation, and validation of Numerical Pain Rating Scale and Global Rating of Change in Tibetan musculoskeletal trauma patients

**DOI:** 10.1038/s41598-024-62777-7

**Published:** 2024-05-25

**Authors:** Jinling Liu, Juncheng Chen, Leilei Tian, Chuan Tang, Wenbin Shuai, Fang Lin, Shilin Luo, Xinxin Xu, Jingjing An

**Affiliations:** 1 Department of Operating Room, West China Hospital, West China School of Nursing, Sichuan University, No. 37 Guoxue Alley, Wuhou District, Chengdu, 610041 Sichuan China; 2 Southwest Minzu University, No. 16 Section 4 First Ring Road, Wuhou District, Chengdu, 610041 Sichuan China; 3 CCU Department of Cardiology, West China Hospital, West China School of Nursing, Sichuan University, No. 37 Guoxue Alley, Wuhou District, Chengdu, 610041 Sichuan China

**Keywords:** Numerical Pain Rating Scale, Global Rating of Change, Translation, Cultural adaptation, Psychometric validation, Challenge, Pain, Health services

## Abstract

Tibetan-speaking patients seeking care in predominantly Mandarin-speaking healthcare settings frequently face communication barriers, leading to potential disparities and difficulties in accessing care. To address this issue, we translated, culturally adapted, and validated the Numerical Pain Rating Scale (NPRS) and the Global Rating of Change (GRoC) into Tibetan (NPRS-Tib and GRoC-Tib), aiming to facilitate cross-linguistic and cross-cultural interactions while examining potential challenges in the adaptation process. Using standard translation-backward translation methods, expert review, pilot testing, and validation through a cross-sectional study with a short-term longitudinal component, we engaged 100 Tibetan patients with musculoskeletal trauma for psychometric validation, including 37 women (aged 22–60 years, mean age 39.1 years). The NPRS-Tib and GRoC-Tib exhibited outstanding psychometric properties, with an Intraclass Correlation Coefficient (ICC) of 0.983 for NPRS-Tib indicating superb test–retest reliability, and expert review confirming good content validity for both instruments. A Spearman's correlation coefficient (Rho) of -0.261 (*P* = 0.0087) revealed a significant, albeit weak, correlation between changes in NPRS-Tib scores and GRoC-Tib scores. The adaptation process also presented notable challenges, including translation discrepancies from translators' diverse backgrounds and levels of expertise, ambiguity in scale options, and the lack of established tools for criterion validity assessment in Tibetan.

## Introduction

Patient-reported outcome measures (PROMs) are pivotal in modern healthcare, providing standardized, objective assessments of clinical outcomes. These tools play a crucial role in reducing bias and variability, thus enabling informed clinical decision-making and facilitating evidence-based practice^1–3^. Among such instruments, the Numeric Pain Rating Scale (NPRS) and Global Rating of Change (GRoC) are widely utilized for their simplicity and effectiveness in capturing patient experiences^4,5^.

The NPRS serves as a subjective measure commonly employed to assess pain intensity. Comprising an 11-point numerical scale ranging from 0 to 10, where 0 indicates no pain at all and 10 indicates the worst possible pain, the NPRS enables a quantitative assessment of pain levels and provides a standardized approach to track changes in pain over time or in response to interventions^6^. The GRoC, on the other hand, is a subjective outcome measure used for assessing a patient's perception of overall change in their health status or a specific health condition, such as pain. It typically involves having patients rate their changes on a scale, which can range from "much worse" to "much better", or employ a numerical scale. The GRoC scale aims to quantify a patient's improvement or deterioration over time, generally to determine the effect of an intervention or to monitor progress during treatment^7^.

In this study, both the NPRS and GRoC were utilized to assess the pain experience. However, the efficacy of these tools could depend on their linguistic and cultural alignment with the patient population, leading to their translation and cultural adaptation into several languages^8–10^.

In China, the Tibetan population, exceeding 7 million, including many within the West China Hospital service area, encounter unique healthcare challenges. Despite high literacy rates and education in Mandarin, a substantial number of Tibetans, especially among the older demographic, may face difficulties communicating in Mandarin within Mandarin-speaking healthcare settings^11–13^. This situation reflects similar challenges encountered by various ethnic and racial groups globally, highlighting the necessity for linguistically and culturally tailored healthcare resources, such as the culturally adapted and validated Tibetan versions of the NPRS and GRoC.

Our study not only focused on the translation, cultural adaptation, and psychometric validation of the NPRS and GRoC into Tibetan but also aimed to explore the methodologies and challenges involved in the process. The choice to adapt these particular tools was influenced by their established usage, the simplicity of implementation, and their adaptability to minority languages such as Tibetan. A significant hurdle in this study was adapting the instruments for cross-language interactions, where health professionals do not speak or understand Tibetan, and the patients lack proficiency in Mandarin. To our knowledge, this is the first formal attempt at translating, culturally adapting, and validating clinical assessment instruments for such a context.

By introducing these tools in the Tibetan language, our goal was to bridge communication gaps, promote health equity, and address the specific needs of Tibetan patients in predominantly Mandarin-speaking areas. Furthermore, the insights gained from this study could highlight potential challenges and solutions, thereby providing a foundation for future efforts to introduce clinical tools in other minority languages.

## Methods and materials

This project was initiated in June 2022 and concluded in early July 2023, including multiple phases.

### Study design

This study employed a cross-sectional design with a short-term longitudinal component to translate, culturally adapt, and validate Tibetan versions of the NPRS and GRoC. We applied the standard translation-backward translation methodology as recommended for clinical assessment instruments^14,15^. The initial translations underwent a systematic cultural adaptation process, followed by validation of the culturally adapted versions to evaluate their psychometric properties.

### Study setting

Our research was conducted in the Trauma Ward of West China Hospital, Sichuan University, a premier tertiary healthcare institution and the foremost general healthcare provider in Southwest China. Serving as a key referral center, West China Hospital draws a diverse patient demographic, notably a significant Tibetan population from the surrounding areas and beyond.

Nonetheless, the proficiency in Tibetan among hospital staff is limited. To aid in communication, Tibetan-speaking volunteer interpreters are sometimes enlisted for complex interactions with patients who are not fluent in Mandarin Chinese. These interpreters, however, are not consistently available for routine care, including pain assessments.

### Participants

#### Enrollment

Tibetan individuals receiving treatment in the Trauma Ward were invited to participate, who were screened based on the following criteria:

Inclusion criteria:

1. Age range: 18–60 years;
2. Ethnicity: self-identified as Tibetan;
3. Language proficiency: capable of understanding written and spoken Tibetan and basic Mandarin Chinese communication;
4. Numerical understanding: ability to count from 0 to 10 in sequence;
5. Pain experience: present pain from a traumatic wound, relatively stable at the study's outset;
6. Cognitive and mental status: no significant known cognitive or mental health conditions.

Exclusion criteria:

1. Age below 18 or above 60 years;
2. Non-Tibetan ethnicity or not self-identified as Tibetan;
3. Inability to read or speak Tibetan or basic Mandarin communication;
4. Inability to count from 0 to 10;
5. Pain from non-traumatic causes or experiencing acute, severe pain with expected significant fluctuations;
6. Significant cognitive or mental health conditions or inadequate cognitive capacity or mental status, as determined by the investigator.

#### Sampling

Our sample size determination was guided by the methodology of Sharma et al*.*, who conducted a similar study on the Nepalese versions^10^, taking into account the desired power and significance levels. A sample size of 100 participants was deemed sufficient.

### Translation and cross-cultural adaptation of NPRS and GRoC

#### Forward translation

The original Chinese versions were independently translated into Tibetan by two bilingual translators. One translator, with healthcare experience and training in pain assessment, aimed for conceptual and semantic equivalence. The other, a language expert without a medical background, ensured linguistic precision.

#### Synthesis of translations

The independent translations were merged into a unified Tibetan version for each instrument. Discrepancies in interpretation, wording, or concept were resolved through discussions among the translators and researchers, reaching a consensus.

#### Back-translation

The consolidated versions were then back-translated into Chinese by two other independent bilingual translators, who were unaware of the original texts.

#### Expert committee review

An expert committee, including the translators, three healthcare professionals with expertise in pain assessment, and a methodologist, was convened. The committee reviewed all versions to confirm semantic, idiomatic, experiential, and conceptual equivalence between the original texts and the translated versions before finalizing the pre-test versions.

Due to potential confusion with the GRoC options, two pre-final versions were created: a comprehensive version with all original options and a simplified version with fewer options, both subjected to pilot testing.

#### Pilot testing

The preliminary versions were tested with a small sample from the target group (20 participants). Participants were asked to complete the scales and provide feedback on each option's clarity, relevance, and acceptability. To gauge comprehension, they were also requested to paraphrase the options in their own words. Based on this feedback and further expert input, minor adjustments were made, resulting in the final Tibetan versions of the NPRS (NPRS-Tib) and GRoC (GRoC-Tib).

During this phase, we also investigated if participants could recall their initial ratings at the time of reassessment to evaluate the impact on test–retest reliability. None were able to recall their precise initial ratings, suggesting minimal risk of recall bias.

When modifying the original instruments to adapt to the Tibetan cultural and linguistic circumstances, we gave careful considerations to maintaining their original intent and validity.

### Validation of NPRS-Tib and GRoC-Tib

For this study, the NPRS-Tib was focused on quantifying pain intensity, and the GRoC-Tib on assessing perceived changes in pain status. The following methods were employed for psychometric validation:

#### Initial assessment with NPRS-Tib

The NPRS-Tib was administered to participants upon their earliest admission to the ward, providing a baseline pain intensity measurement. Participants filled out a printed scale, with a nurse available to clarify any queries without the assistance of family members or an interpreter.

#### Reassessment with NPRS-Tib and GRoC-Tib

After a 2-day interval, participants were reassessed with both the NPRS-Tib and GRoC-Tib, without reference to the initial NPRS-Tib scores. The short interval was chosen to accommodate the rapid turnover and potential changes in pain levels among trauma patients in the ward.

#### Reliability assessment

Test–retest reliability for the NPRS-Tib was evaluated by comparing scores from the initial assessment and reassessment. The Intraclass Correlation Coefficient (ICC 2,1) was calculated, adhering to the guidelines for single measurement, absolute agreement, in a two-way random-effects model.

#### Validity assessment

Content and construct validity for both instruments were assessed to confirm their ability to capture pain intensity levels and perceptions of pain change. Additionally, floor and ceiling effects were analyzed, and the NPRS-Tib's sensitivity to change was evaluated using the Wilcoxon signed-rank test.

Missing values were not encountered due to our comprehensive data collection process, ensuring the completeness of the dataset, including demographic and clinical characteristics and primary outcome measures.

Acceptability was gauged through participant feedback during both pilot testing and validation, focusing on the instruments' comprehension, cultural appropriateness, and content.

Given the single-item nature of both instruments, traditional measures of internal consistency, such as Cronbach's alpha, were deemed inapplicable.

### Statistical analyses

All analyses were conducted using SPSS software (version 23). Descriptive statistics summarized participant demographics and baseline characteristics. Continuous variables were presented as means ± standard deviations (SD), and categorical variables were described using frequencies and percentages.

For test–retest reliability, an ICC value close to 1 indicated excellent reliability, with values above 0.75 considered good. Spearman's rank correlation coefficient was utilized for correlation analysis due to the ordinal nature of the scores. A *p*-value < 0.05 was considered statistically significant.

### Ethical approval and informed consent

The study received ethical approval from the Ethics Committee of West China Hospital, Sichuan University (#WCH2022107). All participants or their legal representatives gave informed consent before participation. The study adhered to the Declaration of Helsinki and followed research guidelines set by national, local, and institutional governing bodies. Patients or the public WERE NOT involved in the design, or conduct, or reporting, or dissemination plans of our research.

### Use of large language model (LLM)

The study's conceptualization, design, data collection, analysis, interpretation of results, and initial manuscript drafting were primarily conducted manually by the authors. ChatGPT-4 (OpenAI, San Francisco, USA) was utilized for English text translation and extensive manuscript proofreading and revision. Responses from ChatGPT were thoroughly reviewed and adjusted to ensure alignment with our intended meanings and standards of writing. The Bing search engine (Microsoft, Washington, USA), enhanced with ChatGPT capabilities, assisted in literature searches, with articles manually reviewed and selected by the authors.

## Results

### Translation and cross-cultural adaption

The translation and cultural adaptation of the NPRS and GRoC into Tibetan turned out to be more challenging than initially expected. Despite their seeming simplicity, achieving usable Tibetan versions of both instruments necessitated several rounds of revision. A significant hurdle was the notable differences between the two translators involved in the forward translation, stemming from their distinct backgrounds and expertise levels. Reconciling their divergent perspectives and linguistic choices to ensure consistent and agreed-upon translations was a complex task.

While the NPRS was successfully translated into Tibetan in its entirety, we encountered issues similar to those outlined by Sharma et al*.*^10^ with the GRoC. Certain options became ambiguous and confusing in the Tibetan translation, as literal translations failed to convey the nuanced gradations present in the original. Expert review further highlighted that the original 15-point GRoC scale was considered "overly extensive," "laden with superfluous options," and "confusing" due to its numerous and unclear options. Consequently, we developed two preliminary versions of the GRoC for pilot testing: a comprehensive version mirroring the original's option count and a simplified version with fewer options. The simplified version condensed the original 15-point GRoC scale into a 7-point scale, spanning from “Much worse” (score − 3) to “Much better” (+ 3).

The decision between the long and short versions was initially unclear, with the former presenting considerable drawbacks, while the latter bore resemblance to the Patient Global Impression of Change (PGIC) scale, another instrument with seven options designed to assess patients' perceived changes in health status^16^. However, pilot testing feedback led to the adoption of the short version. Notably, 65.0% of pilot participants (13/20) described the comprehensive version as “confusing,” “unclear,” or “odd.” Furthermore, the pilot feedback prompted a revision of the estimated sample size to 100 participants.

A small number of pilot participants (4/20, 20.0%) also expressed similar concerns about the preliminary NPRS in Tibetan, though this was ultimately considered minor by the expert panel. This concern was subsequently found to be minimal in the broader validation study, with only sporadic comments or requests for clarification regarding the scale’s range.

### Validation of NPRS-Tib and GRoC-Tib

#### Participant demographics

For our validation study, we recruited 107 Tibetan patients from August 2022 to May 2023. Out of these, seven participants dropped out (6.5%) due to discharge before the reassessment could take place. As a result, a total of 100 participants (63 men, age range 22–60 years, mean age 39.1 years) completed the study. The majority had an education level of junior high school or higher (97, 97.0%). Detailed demographic data are presented in Table 1.

**Table 1:** Demographic characteristics of participants (N = 100).

Characteristic	Participants
Age (years)	
Range	22–60
Mean ± SD	39.1 ± 9.8
Gender	
Male	63 (63.0 %)
Female	37 (37.0 %)
Education	
Primary school	13 (13.0 %)
Junior high school	30 (30.0 %)
Senior high school	35 (35.0 %)
College or over	22 (22.0 %)
Occupation	
Farm work	51 (51.0 %)
Small business owner	11 (11.0 %)
Government employee	14 (14.0 %)
Unemployed	22 (22.0 %)
Others	2 (2.0 %)

*SD* standard deviation.

#### Pain sites and outcomes

Approximately half of the participants reported pain located in the torso (53, 53.0%), with the next most common area being the extremities (33, 33.0%). On reassessment using the NPRS-Tib, the majority demonstrated improvements in pain intensity (81, 81.0%), with 12 showing no change (12.0%) and 7 experiencing worsening pain (7.0%). According to the GRoC-Tib scores, 88 participants reported improvements (88.0%), 5 reported no change (5.0%), and 7 reported worsening pain (7.0%) (Table 2).

**Table 2:** Pain sites and outcomes (NPRS-Tib, GRoC-Tib) (N = 100).

Pain	Frequency (%)	Mean ± SD
Site		
Head & neck	4 (4)	
Extremity	53 (53.0)	
Torso	33 (33.0)	
NPRS-Tib score		
Initial		5.0 ± 1.75
Reassessment		3.15 ± 2.19
Change		−1.85 ± 1.49
GRoC-Tib score		
Much worse (− 3)	1 (1.0 %)	
Worse (− 2)	2 (2.0 %)	
Slightly worse (− 1)	4 (4.0 %)	
No change (0)	5 (5.0 %)	
Slightly better (+ 1)	32 (32.0 %)	
Better (+ 2)	26 (26.0 %)	
Much better (+ 3)	30 (30.0 %)	

#### Reliability

The ICC for NPRS-Tib scores was determined to be approximately 0.983, indicating an excellent level of agreement in pain scores between the initial assessment and reassessment, suggesting high reliability of the NPRS-Tib's over the 2-day interval.

#### Validity

Revisions to the wording and presentation of options, particularly for the GRoC-Tib, were made following feedback from the expert panel. The panel concurred that the final versions of the NPRS-Tib and GRoC-Tib effectively represented the targeted pain-related constructs and were apt for the Tibetan-speaking patient cohort.

Correlation analysis produced a Spearman's correlation coefficient (Rho) of -0.261, signifying a weak inverse relationship between changes in NPRS-Tib scores and GRoC-Tib scores (*P* = 0.0087). The analysis suggested that as pain intensity, as measured by the NPRS-Tib, decreased, the perceived improvement in pain, as assessed by the GRoC-Tib, increased, though the relationship was not markedly strong.

Floor and ceiling effects analysis revealed no instances of ceiling effects for the NPRS-Tib at either the initial assessment or reassessment, indicating the instrument's adequacy in capturing the upper range of pain without clustering scores at the maximum. A floor effect was observed in 13.0% of participants during reassessment, reflecting the instrument's sensitivity to improvements in pain status following clinical intervention.

The Wilcoxon signed-rank test yielded a significant result (*P* < 0.001), indicating a substantial reduction in NPRS-Tib scores from initial assessment to reassessment. This demonstrates the NPRS-Tib's sensitivity to detecting changes in pain levels over time, which affirms its potential utility in clinical settings for monitoring patient progress.

Participant feedback confirmed the acceptability of both the NPRS-Tib and GRoC-Tib, with revisions made post-pilot testing to enhance clarity and cultural relevance. The validation phase participants found the final versions of the instruments understandable and appropriate, with no reports of discomfort or offense.

## Discussion

The communication challenges commonly faced in clinical practice between patients from minority groups and Mandarin-speaking clinicians inspired us to bridge this gap. As an initial step towards introducing established clinical assessment instruments to support cross-language and cross-cultural interactions, we selected the NPRS and GRoC for their outstanding simplicity. This study was driven by two main objectives: first, to translate, culturally adapt, and validate these instruments into Tibetan for clinical use; second, to explore the methodologies and challenges involved in this process, particularly identifying potential pitfalls to inform future initiatives.

Both NPRS-Tib and GRoC-Tib exhibited excellent psychometric properties. The high ICC of 0.983 for the NPRS-Tib underscores its strong reliability, with an excellent level of agreement between initial and reassessment pain scores and stability over a short interval, which is essential for an assessment tool in dynamic clinical settings. Our findings align with those of previous studies, which reported ICC values in the range of 0.72–0.95, highlighting the robustness of these tools across diverse patient populations^4,9,10^.

The content validity, confirmed by the expert panel, demonstrates the cultural and linguistic suitability of both instruments in the Tibetan context. However, the correlation analysis revealed a statistically significant but weak inverse correlation (Rho = − 0.261) between changes in NPRS-Tib scores and GRoC-Tib scores. This indicates that while decreases in pain intensity generally correspond to perceived improvements, the relationship is not as pronounced as expected. This diverges from the reports of Ibrahim et al. and Sharma et al*.* where closer correlations were observed, with *r* = 0.415 and 0.43 respectively^9,10^. This discrepancy could be attributed to individual differences in pain perception and reporting, and cultural factors that influence the expression and interpretation of pain changes.

The high reliability of NPRS-Tib supports its utility in clinical settings for monitoring pain intensity changes over time. However, the weaker correlation suggests caution in using these tools for clinical decision-making. Further studies might be needed to explore how cultural nuances in pain expression and interpretation impact the use and effectiveness of such scales.

Translating and culturally adapting the NPRS and GRoC into Tibetan presented more complexities than anticipated. These seemingly straightforward instruments posed significant challenges in a different linguistic and cultural context. A major issue was the significant variations between translators, which led to considerable challenges in achieving consistency in translations, particularly in the choice of Tibetan terminology for medical terms. It required several revisions to harmonize their translations. A potential strategy for future endeavors might involve modifying the standard translation and back-translation process by employing two multidisciplinary translator teams, each consisting of a healthcare professional and a layperson, working independently. This approach, however, would require further methodological discussion and validation.

Our experience with adapting the GRoC was particularly revealing, echoing the challenges faced by Sharma et al.^10^. This suggests that linguistic discrepancies could be a widespread issue in cross-cultural adaptations of clinical tools, especially for languages spoken by minority groups. Our approach of developing two versions of the GRoC for pilot testing may offer a flexible strategy for similar future projects. The resemblance of the shorter version to the PGIC scale also highlights the potential benefits of leveraging existing, established instruments.

In cross-cultural, cross-linguistic settings, especially where users lack proficiency in each other's languages, creating bilingual versions of instruments might be preferable. This option was indeed contemplated but was ultimately set aside in favor of monolingual versions to avoid complicating the study with the intricacies of bilingual interactions. Since this was our first endeavor in this area, maintaining simplicity was deemed prudent. Nevertheless, the exploration of bilingual instruments in future research is encouraged.

Another significant hurdle was the absence of pre-existing, validated instruments in Tibetan for criterion validity assessment. This issue, while not unique to our study, is a common challenge in research involving minority languages and impedes comprehensive validity testing. As a result, we resorted to alternative forms of validity testing, such as construct and content validity, although these do not fully replace criterion validity. This highlights the pressing need for more research into developing clinical assessment tools in minority languages.

### Limitation

While this study offers significant insights into the adaptation and validation of clinical assessment tools in Tibetan, it has limitations. A primary limitation was the inability to conduct a criterion validity assessment, attributed to the absence of established, validated instruments in the Tibetan language. It is imperative for future research to incorporate criterion validity assessments as comparable instruments become available in Tibetan. Another limitation was our selection of trauma patients to assess the test–retest reliability of the NPRS-Tib. This particular patient demographic is notable for the rapid fluctuations in pain levels over short periods, which could potentially influence the reliability scores. Despite our proactive measures to minimize this confounding effect by opting for a brief 2-day interval and carefully selecting patients with relatively stable pain conditions, this remains a consideration. In subsequent research, exploring different patient groups may provide a strategy to further mitigate this risk.

## Conclusions

The Tibetan versions of the NPRS and GRoC are effectively translated and culturally adapted. Both NPRS-Tib and GRoC-Tib demonstrate outstanding psychometric properties, making them appropriate for use in clinical contexts. The experiences gained from the translation, cultural adaptation, and validation processes offer valuable lessons for future similar endeavors, including navigating translation challenges from divergences among translators, addressing the ambiguity of scale options in Tibetan, and compensating for the absence of established benchmarks necessary for criterion validity assessment.

## Data Availability

The data analyzed in our study are available on reasonable request to the corresponding author. Notably, to read the digital versions of NPRS-Tib and GRoC-Tib on a PC, a special Tibetan font is needed, which is not available on MS Word program by default and not available on smartphones or portable devices such as PDAs commonly used in clinical setting. The instruments can be provided on reasonable request to the corresponding author.
